# Evidence-based practices for prevention and management of medical device alarm fatigue in anesthesia professionals

**DOI:** 10.3389/fmed.2025.1551222

**Published:** 2025-08-18

**Authors:** Shaoru Chen, Jie Wang, Hui Zhi, Hongmei Zhang

**Affiliations:** ^1^Department of Anesthesia and Perioperative Medicine, Henan Provincial People’s Hospital, Zhengzhou, China; ^2^Henan Provincial Key Medicine Laboratory of Nursing, Zhengzhou, China; ^3^Zhengzhou University People’s Hospital, Zhengzhou, China; ^4^Henan University People’s Hospital, Zhengzhou, China; ^5^Department of Nursing, Henan Provincial People’s Hospital, Zhengzhou, China

**Keywords:** alarm fatigue, anesthesiology, evidence-based practice, management, prevention

## Abstract

**Objective:**

To assess efficacy and implement the best available evidence for managing and preventing alarm fatigue in a healthcare context.

**Methods:**

Four phases of evidence acquisition, status review, evidence application, and effect evaluation were used to apply evidence-based practice to medical care in the PACU between January and June 2024. Prior to and following the application of evidence, the occurrence of unfavorable outcomes pertaining to the management of surgical patient monitors and anesthesia alarm reports, the degree of evidence-based organizational culture, the implementation rate of review indicators, and the associated knowledge level of alarm fatigue prevention and management were all compared.

**Results:**

Following the implementation of evidence-based practice, the indicators related to Anesthesiologists and nurses were reviewed, and the implementation rate was improved compared with the baseline. The frequency of negative outcomes associated with surgical patient monitoring and anesthesia alarm management was considerably decreased. Anesthesiology physicians’ and nurses’ knowledge of alarm fatigue management (t = −7.027, *p* < 0.001) and evidence-based practice skills (t = −52.804, *p* < 0.001) improved. The degree of evidence-based organizational culture was higher than baseline (t = −23.864, *p* < 0.001), while clinical alarm fatigue (t = 37.454, *p* < 0.001) and barriers to evidence-based nursing practice (t = 41.508, *p* < 0.001) were lower than baseline.

**Conclusion:**

Continuous quality improvement is still required in subsequent clinical settings, but evidence-based practice in the Department of Anesthesiology and Perioperative Medicine can effectively standardize the alarm practice behaviors of healthcare professionals, enhance evidence-based competence, and lower the incidence of patient-related adverse events in alarm management.

## 1 Introduction

Medical device alarm fatigue refers to a phenomenon in which healthcare workers are exposed to a large number of medical device alarms for an extended period of time, become less sensitive to medical device alarms, and thus ignore or delay responding to device alarms (1). Advances in medical equipment have been made with modern technology, making hospital environments noisier while also providing patients with more convenient care (2). Early warning systems have a high risk of causing “alarm fatigue,” where personnel ignore alarms because they believe there are too many false alarms (3). Alarm fatigue is a serious problem faced by many healthcare workers, according to alarm management guidelines released in 2020 (4). According to The Joint Commission and the American Association of Nurse Anesthesiologists, alert tiredness is a serious problem that has to be addressed urgently. It is strongly tied to patient safety and the standard of care (5, 6). “Strengthening the safety and alarm management of medical equipment” was included for the first time as one of the top ten goals in the Patient Safety Goals (2019 Edition) published by the China Hospital Association (7). The current issues include medical staff members’ ignorance of alert risk prevention, the absence of a framework for safety training and evaluation of medical equipment use, and a lack of initiative in reporting adverse events related to devices. Alarm dangers have consistently been listed among the top ten health technology hazards by the global organization, Emergency Care Research Institute (ECRI). These risks include failing to respond to alarms appropriately or setting them incorrectly; however, alarm fatigue was not recognized as a significant issue until 2020 (8, 9). It can cause healthcare professionals to experience irritation and aggravation as well as memory loss, improper override reactions, delayed processing of alerts, and diminished recall of alarms (10). Medical alerts are mostly used by nurses who face a complex work environment, a large volume of alerts, and frequent adverse events linked to them (11). The Alert Security Handbook: Strategies, Tools, and Guidelines (12), released by the ECRI, offers hospitals a framework for developing in-house alert management initiatives. Adverse nursing events that are given much attention include pressure injuries, falls/bed falls, prescription errors, and slipped lines; whereas adverse events connected to alarms are currently given less focus.

Alarm creation, alarm transmission, alarm recognition, and alarm response are the four phases of alarm management. Alarm fatigue can be successfully decreased with targeted actions at every step (4). Practice guidelines (4), evidence summaries (13–15), and systematic evaluations (16, 17) related to alarm fatigue management have been published both nationally and internationally, and there is a greater basis for evidence-based practice. An evidence-based interdisciplinary team was assembled by Srinivasa et al. (16) to offer a useful approach for assessing and treating cardiac telemetry alarm fatigue. In order to establish norms and quality control standards for the prevention and management of alarm fatigue in this specialty area, this study applies the best available evidence to anesthesiology providers who perform these tasks in the Department of Anesthesiology and Perioperative Medicine. It does this by translating and applying the evidence through a baseline review and evidence application. The objective of this study was to lower the frequency of unfavorable outcomes related to anesthesia machine and patient monitor alerts by using an evidence-based clinical application to enhance organizational culture, alert knowledge, evidence-based practice, and health care delivery. This study was carried out at the BPSO Direct Application Center, JBI Evidence Based Nursing Collaborative, which has finished the institution’s Evidence Based Practice Program Filing (2024). ES20233885 is the registration number.

## 2 Methods and subjects

### 2.1 Subjects

The anesthesia recovery room of the Department of Anesthesiology and Perioperative Medicine at a tertiary-level general hospital in Henan Province serves as the practice site. The evidence application scenario is evaluated because the average number of patients admitted to the anesthesia recovery room each year exceeds 60,000, many of whom require instrumentation monitoring following general anesthesia, and nurse anesthetists’ alarm fatigue is high. Additionally, the department staff regularly works the anesthesia machines, monitors, and other equipment, and the anesthesia nurse posts regularly set up instrumentation management teams. The department’s employees frequently operate monitors, anesthetic machines, and other tools and equipment, which exposes them to high levels of risk. Technical assistance for the extraction of adverse events related to alarms can be obtained from the department’s well-established adverse event management platform.

Patients recovering from general anesthetic and anesthesiology nurses who prevented and managed alarm tiredness served as the study’s subjects. Patient inclusion criteria: (i) age ≥18 years old, (ii) American society of Aneshesiologists (ASA) classification II to III, (iii) transferred to the anesthesia recovery room of our hospital after general anesthesia surgery. Exclusion criteria: (i) those who were transferred to ICU unplanned during resuscitation, (ii) those who had poor communication and language barriers.

Inclusion criteria for nurses: (i) anesthesiology department working time ≥1 year, (ii) junior or above title, (iii) a college degree or higher in order for nurses to comprehend and adhere to the implementation of evidence-based nursing practice. Exclusion criteria: (i) members within the study group, (ii) nurses who transferred to other departments and advanced training.

### 2.2 Methods

This study conducted an evidence application program using the Joanna Briggs Institute’s (JBI) Best Evidence Clinical (18). The program was based on a four-step process for implementing evidence: obtaining evidence, baseline review before evidence application, evidence-based clinical application and effectiveness evaluation.

#### 2.2.1 Phase I: obtaining evidence

##### 2.2.1.1 Determining the research questions

The PIPOST approach (19) of Fudan University’s Center for Evidence-Based Nursing was used to create evidence-based questions. P (Population) is the clinical nurse who applies the monitoring device. I (Intervention) for alarm management programs: including environmental and personnel preparation, multidisciplinary collaboration, defining threshold ranges and prioritization of alarms, regular assessment and feedback, reducing the incidence of false alarms, education and training, and the use of smart technologies. P (Professional) is a multidisciplinary professional. O (Outcomes) is a change in alarm management workflow for monitoring devices on the system side; a reduction in alarm fatigue on the practitioner side by working according to an alarm management program; and a reduction in the incidence of adverse outcomes related to monitoring devices and anesthesia machines on the patient side in the three areas of alarm generation, alarm recognition, and alarm response. S (Setting) is Post anesthesia care unit. T (Type of evidence) for clinical practice guidelines, evidence summaries, expert consensus, systematic evaluations, RCTs.

##### 2.2.1.2 Search strategy

Top-down evidence search based on the “6 s” evidence model (13). Retrieve computerized decision-making systems, including Up to date. The guideline networks included Medical Pulse, National Institute for Health and Clinical Excellence (NICE), Scottish Intercollegiate Guidelines Network (SIGN). Databases included searching Cochrane Library, PubMed, Web of Science, Embase, CINAHL, Sino Med, China National Knowledge Infrastructure (CNKI) and Wanfang databases. Professional society websites include the Emergency Care Research Institute, ECRI website, The Joint Commission (TJC), AAMI Foundation alarm resources, National Association of Clinical Nurse Specialists Alarm Fatigue website. The timeframe of the search was from the establishment of the database to December 31, 2023 for all articles. Twenty-five articles were included by reading the full text, including 4 guidelines, 1 expert consensus, 3 evidence summaries, 14 systematic evaluations, 1 national standard, and 2 RCTs.

##### 2.2.1.3 Assessment of the evidence

Appraisal of guidelines for research and evaluation II (AGREE II) was used to evaluate the included guidelines (20). Evidence summaries were assessed by going back and reviewing the original text. The JBI Evidence-Based Health Care Center Systematic Evaluation Criteria (2016) were used to assess the systematic evaluation (21, 22). The JBI Center for Evidence-Based Health Care (2016) Authenticity Assessment Tool for Opinion and Consensus Articles was used to assess expert consensus (23). In seven areas—environmental and personnel readiness, multidisciplinary collaboration, threshold range definition and alarm prioritization, frequent evaluation and feedback, lowering the number of false alarms, education and training, and smart technology use—this produced 34 pieces of evidence.

##### 2.2.1.4 Creation of review indicators

The most pertinent individuals in clinical nursing practice, such as anesthesiologists, anesthesiology nurses, were gathered to use an expert meeting technique to assess the practical applicability of the findings (24). Through the expert meeting, two clinical nursing experts and two evidence-based experts applied the FAME framework, namely feasibility, appropriateness, meaningfulness, effectiveness, the 34 pieces of evidence were evaluated through these four aspects (25). According to the evidence’s applicability evaluation, the PACU is the implementation environment, a significant number of monitoring alerts are generated, the evidence items are helpful for the patients’ pertinent outcomes, and the feasibility is strong. Because of the high cost of IoT and the impossibility of interdisciplinary data collecting at the hospital level, it was decided at this stage to exclude five pieces of evidence: (i) hospitals should create alarm management policies, procedures, and systems. (ii) Clinical leaders should prioritize the safety of the hospital’s alarm systems. (iii) Interprofessional teams should gather data related to alarms and utilize that data to inform alarm management decisions. (iv) To manage “alarm rounds,” nursing administrators create policies and procedures that ensure that only patients with clinical indications are monitored or immediately removed from surveillance. (v) Information technology, including Internet of Things (IoT) technologies, is used by hospitals to combat alarm fatigue. In addition, 3 pieces of evidence were in line with the current clinical application status, with an implementation rate of 100%, and will not be transformed: To increase adherence to alarm modifications, nurses are recommended to note alarm parameters in the patient’s medical file. It is advised to evaluate the integrity of the electrodes and leads, utilize disposable cardiac lead wires, and change the electrode pads every 24 to 48 h or whenever the ECG is not detected well. The bedside nurse positions and utilizes the SpO_2_ probe correctly. Based on the team’s previously created summary of the best evidence for the prevention and management of medical device alarm fatigue in clinical nurses, 26 pieces of evidence were finally kept to create 30 review metrics. Members of the Evidence Clinical Application Panel used the Adverse Outcome Checklist Related to Surgical Patient Monitor and Anesthesia Machine Alarm Management, as well as the Review Form for Prevention and Management of Medical Device Alarm Fatigue for Nurse Anesthetists, to perform the review (Table 1).

**Table 1:** Evidence, review indicators for anesthesia nurses’ alarm fatigue management and prevention.

Subject of evidence	Content of evidence	Review of indicators
Environment and personnel preparation	Evidence 1: Enhancing hospital noise environments and implementing noise reduction techniques (38, 39)	Indicator 1: There is noise monitoring equipment in the area, and the alert volume is adjusted properly. Indicator 2: The department implements noise reduction techniques.
	Evidence 2: The instrument and equipment operate normally with no mechanical vibration or electromagnetic interference from the surroundings (40)	Indicator 3: The department’s atmosphere is free of electromagnetic interference and mechanical vibration, which can interfere with instruments and equipment operating normally.
	Evidence 3: Clinical alert management to increase nursing care safety by adjusting workload completely, including how human resources are allocated and how the alert is set (38, 39)	Indicator 4: According to the National Health Office Medical Letter [2019], document No. 884, which is the “Notice of the General Office of the National Health Commission on the Issuance of Guidelines for the Construction of Anesthesiology Medical Service Capacity” the Department distributes personnel.
Multidisciplinary cooperation	Evidence 4: To address alert management, hospital and nursing administration should form an interdisciplinary team or group (4, 14, 15, 17, 41), conduct an alert risk assessment, and explore alert reduction strategies (17, 39)	Indicator 5: creation of interdisciplinary alarm management teams in departments and interdisciplinary alarm management committees in hospitals
Define threshold ranges and prioritize alerts	Evidence 5: Recommendation to rationalize alarm parameters and change thresholds (42, 43), It is advised to consult the most recent standards of practice for ECG monitoring in hospitals published by the American Heart Association and to adjust the monitoring parameters’ alert thresholds to the average patient monitoring value ± (20 to 30%)	Indicator 6: The average patient monitoring value ± (20–30%) is the alarm threshold that nurses set for monitoring parameters.
	Evidence 6: Suggest that medical facilities make clear how alerts are categorized and graded, establish guidelines for handling and responding to alerts, and determine which alerts in each specialty need to be treated first (4). Determine the quantity of alarms, the source of the alarm-generating device, and the clinical importance of the alerts based on their severity (10, 44, 45), Answer the alarm within the time limit (4). Critical Alerts set to Critical/High Superior Alerts for recommended arrhythmia (cardiac arrest, ventricular fibrillation, and pulseless ventricular tachycardia) (15, 18)	Indicator 7: Department clear alerts go into one of three priority categories: high, medium, or low. Indicator 8: Response times for various warning levels are explained in this section.
	Evidence 7: Assess the alarm parameter settings in light of the patient’s medical needs, hospital or departmental policies, and medical advice. Encourage nurses to dynamically modify the alarm parameter settings within predetermined bounds (10, 39, 41, 43), Establish alarm parameters that minimize inoperable alarms while maintaining the safest monitoring level (17, 45, 46)	Indicator 9: Nurses evaluate alarm parameter settings and make dynamic adjustments.
	Evidence 8: The alarm volume should be adjusted based on the daytime/nighttime period, the alarm level, and the surrounding noise level. Establish volume alert limitations at low priority first, then raise it to medium level if the situation persists (14); as needed, night shift nurses adjust the volume of instrument and equipment alarm sounds based on the hospital’s or unit’s system and the patient’s condition (13, 14)	Indicator 10: The alert volume is changed by nurses based on the circumstances.
Regular evaluation feedback	Evidence 9: Test the instrument’s performance evaluation, being sure to check the safety alarm feature (40); examine and modify the alarm default settings provided by the manufacturer to suit the community under observation (17)	Indicator 11: Nurses evaluate how well instruments work. Indicator 12: Nurses modify alarm settings based on patient status.
	Evidence 10: Encourage nurses to evaluate patients’ risks (38, 47)	Indicator 13: Nurses evaluate a patient’s condition’s risk.
	Evidence 11: Timing and content of alarm assessment: Alarm settings are checked at the beginning of each shift, at the time of a change in the patient’s condition and at the time of a change in the nurse’s condition, in accordance with departmental or hospital policy and the patient’s condition, and alarm parameters, on/off status and alarm delay settings are assessed (4, 41, 42, 48)	Indicator 14: Nurse evaluation of the alarm delay settings, on/off status, and alarm parameters
	Evidence 12: It is advised that nurses’ levels of alarm fatigue be measured using the Subjective Fatigue Symptoms Scale (4)	Indicator 15: Using the Fatigue Symptoms Scale for assessment
Reduces the incidence of false alarms	Evidence 13: Improve signal conductivity and lower the amount of false alarms by using electrode pads and properly prepping the skin (4, 13, 17, 39, 41, 45). Cleaning the skin where the electrode pads are applied with alcohol is not advised (4)	Indicator 16: Nurses’ appropriate skin preparation Indicator 17: Nurses’ appropriate electrode use
	Evidence 14: Reduce patient-operated alarms that are disregarded or ineffectual by using short alarm delay strategies (e.g., heart rhythm alarm delay of no more than 10s; 15–30s without exception) prior to the delivery of specialized care (39, 43, 45)	Indicator 18: The brief alarm delay technique used by nurses
Education and training	Evidence 15: It is advised that care managers create unit-specific default parameters and alarm management guidelines (41). Implementing interdisciplinary education, evaluating nurses’ alarm management proficiency, creating training programs, creating alarm management processes, and incorporating clinical nurses’ feedback into the creation and execution of alarm management policies and procedures (38) to reduce alarm fatigue among nurses (2, 10, 44, 49, 50)	Indicator 19: Default settings and policies for handling anesthetic unit alerts are developed departmentally.
	Evidence 16: Take part in the creation of the alert procedure, read and discuss articles about the detrimental effects of alarm fatigue every week, and research the most effective alert management techniques (50)	Indicator20: Every week, nurses read and debate articles about alarm fatigue and investigate the best alarm management techniques.
	Evidence 17: It is advised that when new members join the organization, when new equipment or technology is introduced, or when alarm management protocols are developed or updated, nursing administrators should give initial and continuing training to hospital and unit staff regarding monitoring systems and alarm management (17, 41), and that the effectiveness of the training be evaluated on a regular basis (4, 49). For aspiring nurses and new nurses, alarm handling training is mandatory. There is a required training program in place for new hires or young nurses, and it is revised frequently in response to technological advancements (13, 49) 6/4/25 8:10:00 PM	Indicator 21: Nurses’ initial and continuing education regarding the unit’s alarms
	Evidence 18: Evidence-based research, expert opinion, and equipment instruction manuals should all be considered when developing training materials. This includes information on specialized knowledge, alarm management systems, and protocols, as well as standards of practice for cardiac monitoring, the goal of the monitoring, different types of alarms, alarm prioritization, electrode pad placement and skin preparation, monitor connection, alarm set-up, identifying clinically important alarms and customizing alarm parameters, alarm response, equipment maintenance, and health education (4)	Indicator 22: The department creates alert training materials.
	Evidence 19: It is advised that nurses of varying seniority receive phased and focused instruction in alerts and daily management based on their individual traits, training background, job experience, and degree of weariness (4, 11) to improve their ability to assess patients’ risks (38)	Indicator 23: The department creates daily management and focused alert training.
	Evidence 20: It is advised that various training techniques, such as lectures, in-person live demonstrations, training during clinical exams, online training, case studies, and simulations, be employed depending on the financial and human circumstances (11, 39), and self-paced learning (15). It is advised to do initial face-to-face training and regular follow-up online training (4)	Indicator 24: Department approaches training in a different way.
	Evidence 21: Suggest that professionals such as clinical care managers, nurse educators, cardiologists, nurses, information engineers, equipment engineers, and hospital safety management experts be involved as trainers in the management of clinical alerts (4)	Indicator 25: Experts who serve as trainers in the Department’s clinical alert management
	Evidence 22: Suggest utilizing a nursing cadre training approach to cultivate alarm co-finishers within the unit who can act as advocates for alterations in alarm management procedures. Additionally, assist in creating a safety culture within the alarm management department and implementing evidence-based practices within the unit by providing real-time evaluations, feedback, and guidance to unit members (4)	Indicator 26: Installation of modular alarm linkers inside the area
Use of smart technology	Evidence 23: Adopting “smart” warning systems is advised in order to increase technical accuracy and decrease technical false alarms. These systems should take into account a variety of characteristics, rates of change, and signal quality (39, 47, 51, 52)	Indicator 27: The department has implemented a “smart” alarm system that considers a variety of factors, including signal quality, rates of change, and parameters.
	Evidence 24: suggests using an integrated alert notification system that offers closed-loop communication and contextual data (39, 43) and changes in the presentation of alarms (43) to reduce the sound load (41, 47)	Indicator 28: Department Alert Integrated Notification System for closed-loop communications and background data
	Evidence 25: Reduce the quantity of sirens that healthcare personnel need to become familiar with by standardizing the sound of alarms (39)	Indicator 29: Alarm sound standardization by department
	Evidence 26: On monitoring equipment, alarm troubleshooting processes are configured to guarantee the equipment’s optimal performance (39)	Indicator 30: Procedures for troubleshooting department monitoring equipment alarm setup

#### 2.2.2 Phase II: baseline evaluation before evidence application

There are eleven members of the evidence-based practice team: three master’s degree students with systematic evidence-based training who are in charge of the clinical application of evidence, five anesthesia recovery room team leaders who are in charge of data collection, an MD and a nurse practitioner who manage and coordinate change, and a PhD in nursing who guides and oversees the application of evidence.

In March 2024, a baseline evaluation was conducted on 30 patients who satisfied the requirements for cardiac monitoring and anesthetic machine instrumentation following general anesthesia, as well as 32 healthcare professionals in the Department of anesthetic and Perioperative Medicine. The anesthetic recovery room team leader, part-time research nurse, and nurse manager were among the members of the Evidence Application Team who assessed each review indicator according to the previously mentioned review indicators and assessed each one’s performance.

#### 2.2.3 Phase III: evidence-based clinical application

Barriers and facilitators to the use of evidence are examined independently, and solutions for improvement are created.

Obstacle factor 1 and countermeasure: the agency lacks management mechanisms and operational protocols for managing and preventing alarm fatigue. Countermeasures include: (i) Create an alert management procedure based on clinical practice, instrumentation manuals, and the best available data. (ii) Enhance and revise alarm fatigue management paperwork, such as creating an implementation guidebook, assessment forms, and educational materials. (iii) An alarm management quality control group was formed, with the department director and head nurse serving as the group leaders. The PACU quality control staff oversaw the alarm management and nursing record content daily and summarized it in the nursing workgroups and the evidence-based group. Additionally, alarm management and a summary of adverse results were added to the weekly quality control content. (iv) Standardize the Department of Anesthesiology’s medical and nursing alarm regulations, and record movies showing how the department’s tools and equipment work for training and education purposes.

Obstacle factor 2 and countermeasure: lack of systematic education and training on alarm management in the department. Countermeasures include: (i) Assemble team members to collaborate on creating a methodical alarm management training program that complements the department’s current instrument and equipment manuals. (ii) Boost instruction in knowledge and abilities linked to managing and preventing alarm fatigue. (iii) In the first place, give the recovery room teams in the Department of Anesthesiology and Perioperative Medicine unified training, train PACU nurses and anesthesiologists in evidence-based knowledge, and then have them follow up with their team members. (iv) Training material was centered on the four alert life cycles, and instruction took place twice a week. Each instruction was followed by a theoretical analysis.

Obstacle factor 3 and countermeasure Countermeasures: inadequate evidence-based capacity of health care. Countermeasures include: (i) Create training programs pertaining to evidence-based practice and invite hospital nursing experts to present in order to raise healthcare professionals’ awareness and proficiency in the area of evidence-based nursing practice of alarm fatigue management. (ii) The evidence application team periodically verifies at random how healthcare professionals are applying evidence in the recovery room. By comparing the findings of the nursing records in the recovery room with the practice’s content, we will give the departmental administrators the results and feedback and quickly change the anesthesiology nurses’ alert operational behavior.

Obstacle factor 4 and countermeasure Countermeasures: inadequate provision of intelligent equipment such as alarm integration. Countermeasures include: (i) Clinical quality improvement initiatives are supported by department directors and nurse managers. (ii) The installation of more large screen monitors improves alarm presentation, lowers sound load, and centralizes the display of vital indicators from electrocardiographic monitoring device. (iii) Ten more assistant nurses have been hired in an effort to reduce the strain of clinical nursing duties.

#### 2.2.4 Phase IV: effectiveness evaluation

Following the application of the evidence, 30 patients who were admitted to the PACU for instrumentation monitoring following general anesthesia and 32 anesthesia practitioners were included once more for a re-examination in June 2024.

Organizational level. The degree of organizational culture was assessed using the Organizational Culture Building Scale for Evidence-Based Practice, which was created by Melnyk et al. (26), which has a Cronbach’s a coefficient of 0.97.

Practitioner level. (i) Based on the Knowing, Believing, Acting model and the features of the clinical alert lifecycle as a theoretical basis, Zou et al. (27) used the nurses’ clinical alert knowledge, attitude, and behavior questionnaire, which has a Cronbach’s a coefficient of 0.88 and can be used to assess anesthesia nurses’ capacity to manage clinical alerts. (ii) Higher total scores indicate higher levels of alarm fatigue. The Alarm Fatigue Scale (AFS), which has seven entries and is scored on a 5-point Likert scale, was used. It was revised by Cho et al. (28), which has a Cronbach’s a coefficient of 0.78. (iii) Nurses’ opinions of variables that hinder evidence-based practice were measured using the Barriers to Evidence-Based Nursing Practice Scale, which was created by Funk et al. (29). A 5-point Likert scale was used to rate the scale, and its Cronbach’s alpha coefficient was 0.919 (30). (iv) The evidence-based nursing competency of anesthesiology nurses was assessed using the Evidence-Based Nursing Competency Scale, which was created by Wang et al. (31). It has a Cronbach’s alpha coefficient of 0.951 overall and uses a 5-point Likert scale, with higher scores denoting stronger evidence-based nursing competency.

Patient level. An alarm management record sheet for surgical patients was used to measure the frequency of unfavorable outcomes associated with the management of monitor and anesthetic machine alarms in terms of alarm generation, alarm recognition, and alarm reaction. (i) Equipment operation, lead wire connection, threshold setting, and alarm on are all included in alarm generating. (ii) Message display is part of alarm recognition. (iii) Technical functioning and alarm awareness are components of alarm response.

### 2.3 Statistical methods

The data was analyzed using SPSS 25.0. The x^2^ test and Fisher’s exact probability test were used to compare the measurement data, which was expressed as mean ± standard deviation, and the count data, which was expressed as frequency and percentage (%).

## 3 Result

### 3.1 General information on the subjects

Thirty PACU nurses and two anesthesiologists made up the 32 medical and nursing staff members who took part in the entire trial, along with the titles of one deputy chief physician, one attending physician, thirteen nurse practitioners in charge, fourteen nurse practitioners, and three nurses.

There were 60 postoperative patients under general anesthesia in all, 25 of whom were men and 35 of whom were women. The 60 patients were split into baseline and evidence-based practice review groups depending on the length of hospitalization before and after evidence application. There was no significant difference in the general data between the two groups, which excluded the bias caused by sample selection, as shown in Table 2.

**Table 2:** Comparison of patients’ general information before and after evidence application (*n* = 60).

Item	Category	Baseline (*n* = 30)	Evidence-based practice (*n* = 30)	t	*p*
Sex	Male	15 (0.5)	10 (0.17)	1.714	0.189
	Female	15 (0.5)	20 (0.83)		
Age		56.57 ± 13.73	52.93 ± 19.80	0.826	0.412
Educational level	Below junior high school	20 (0.67)	20 (0.67)	0.202	0.904
	High school	6 (0.2)	5 (0.17)		
	University degree and above	4 (0.13)	5 (0.17)		
Marital status	Married	29 (0.97)	24 (0.8)	4.043	0.108
	Unmarried	1 (0.03)	6 (0.2)		
Type of anesthesia	General anesthetic	8 (0.27)	10 (0.33)	0.317	0.573
	General anesthesia + nerve block	22 (0.73)	20 (0.67)		
ASA	II	5 (0.17)	4 (0.13)	0.131	0.718
	III	25 (0.83)	26 (0.87)		

### 3.2 Comparison of the implementation of the review indicators before and after the application of evidence

The on-site review tool “Evidence-based nursing practice checklist for the prevention and management of alarm fatigue” was used to review the operations related to the medical and nursing level before and after the application of evidence, and the implementation rate was improved compared with the baseline, and the difference was statistically significant. System-level review indications 1 through 5 had a 100% implementation rate, followed by indicator 7 through indicator 8, indicator 15, indicator 19, and indicator 21 through indicator 30 (see Table 3).

**Table 3:** Comparison of the implementation of indicators related to anesthesiologists and nurses before and after the application of evidence (%).

Review of indicators	Baseline (*n* = 32)	Evidence-based practice (*n* = 32)	*x^2^*	*p*
Indicator 6	15 (46.88)	29 (90.63)	14.255	<0.001
Indicator 9	5 (15.63)	23 (71.88)	20.571	<0.001
Indicator 10	7 (21.88)	28 (87.5)	27.807	<0.001
Indicator 11	1 (3.13)	14 (43.75)	14.716	<0.001
Indicator 12	2 (6.25)	22 (68.75)	26.667	<0.001
Indicator 13	15 (46.88)	32 (100)	21.149	<0.001
Indicator 14	14 (43.75)	28 (87.5)	13.576	<0.001
Indicator 16	16 (50)	30 (93.75)	15.150	<0.001
Indicator 17	12 (37.5)	30 (93.75)	22.442	<0.001
Indicator 18	3 (9.38)	28 (87.5)	39.101	<0.001
Indicator 20	1 (3.13)	23 (71.88)	32.267	<0.001

The content of review indicators as shown in Table 1.

### 3.3 Comparison of the incidence of adverse outcomes associated with the management of monitor and anesthesia machine alarms in surgical patients before and after the application of evidence

The frequency of unfavorable outcomes pertaining to the handling of anesthesia machine and monitor alarms in surgical patients was used to assess patient-level implementation outcomes (Table 4). Compared to the baseline review phase, the incidence of adverse outcomes related to the management of both monitors and anesthesia machines decreased when evidence-based practices for alarm fatigue management were implemented. There were statistically significant changes in threshold settings (25 vs. 14, x^2^ = 8.864, *p* < 0.05), technical practice (15 vs. 1, x^2^ = 11.167, *p* < 0.05), and monitor alarm awareness (23 vs. 11, x^2^ = 9.774, *p* < 0.05) following implementation. There were statistically significant differences in respiratory circuit connection (9 vs. 3, x^2^ = 3.750, *p* < 0.05), alarm on (9 vs. 3, x^2^ = 3.750, *p* < 0.05), information display (11 vs. 2, x^2^ = 7.954, *p* < 0.05), technical operation (10 vs. 3, x^2^ = 4.812, *p* < 0.05) and alarm consciousness of anesthesia machine (9 vs. 2, x^2^ = 10.276, *p* < 0.05).

**Table 4:** Ratio of incidence of adverse outcomes related to monitor and anesthesia machine alarm management before and after evidence application (%).

Instrumentation	The chain of events	Relevant factor	Baseline (*n* = 30)	Evidence-based practice (*n* = 30)	*x^2^*	*p*
Monitor (device)	Alarm Generation	Equipment operation	16 (53.3)	10 (33.3)	2.443	0.118
		Lead wire connection	10 (33.3)	5 (16.7)	2.222	0.133
		Threshold setting	25 (83.3)	14 (46.7)	8.864	0.002
		Alarm on.	9 (30)	4 (13.3)	2.455	0.113
	Alarm recognition	Information display	13 (43.3)	15 (50)	0.268	0.605
	reply to an alarm	technical operation	15 (50)	1 (3.3)	11.167	0.002
		Alert Awareness	23 (76.7)	11 (36.7)	9.774	0.001
Anesthesia machine	Alarm Generation	Equipment operation	8 (26.7)	4 (13.3)	1.667	0.193
		Breathing line connection	9 (30)	3 (10)	3.750	0.049
		Threshold setting	8 (26.7)	5 (16.7)	0.884	0.345
		Alarm on.	9 (30)	3 (10)	3.750	0.049
	Alarm recognition	Information display	11 (36.7)	2 (6.7)	7.954	0.003
	reply to an alarm	technical operation	10 (33.3)	3 (10)	4.812	0.025
		Alert Awareness	9 (30)	2 (6.7)	10.276	0.004

### 3.4 Comparison of anesthesiology provider alarm fatigue management knowledge levels, clinical alarm fatigue, barriers to evidence-based nursing practice, and evidence-based practice competencies before and after evidence application

In the post-evidence application review, anesthesiology providers’ knowledge, attitudes, and current behavioral status questionnaire scores on managing and preventing alarm fatigue were higher than in the pre-evidence application, and the difference was statistically significant (t = −7.027, *p* < 0.001), and the score increased significantly after the intervention (82.53 vs 90.59, *p* < 0.001). The difference between the Alarm Fatigue Scale scores before and after the application of the evidence was statistically significant (t = 37.454, *p* < 0.001), and the score decreased significantly after the intervention (31.75 vs. 14.69, *p* < 0.001). Scores on the Barriers to Evidence-Based Nursing Practice Scale were statistically significant lower than they were prior to the use of the evidence (130.63 vs. 72.59, t = 41.508, *p* < 0.001). Prior to the use of evidence, scores on the Evidence-Based Competency Questionnaire were lower, this difference was statistically significant (5.66 vs. 67.25, t = −52.804, *p* < 0.001), as shown in Table 5.

**Table 5:** Comparison of the level of knowledge and evidence-based practice of fatigue prevention and management in anesthesiology healthcare alerts before and after the application of evidence (x̅ ± s, *n* = 32).

Item	Baseline	evidence-based practice	*t*	*p*
Total score for building an evidence-based organizational culture in hospitals	39.53 ± 7.62	82.63 ± 2.67	−23.864	<0.001
Total Score of Knowledge, Trust and Conduct	82.53 ± 6.18	90.59 ± 2.72	−7.027	<0.001
Alarm fatigue level	31.75 ± 3.24	14.69 ± 0.82	37.454	<0.001
Alerting to barriers to practice	130.63 ± 12.30	72.59 ± 5.33	41.508	<0.001
Evidence-based capacity	5.66 ± 7.77	67.25 ± 1.65	−52.804	<0.001

### 3.5 Comparison of organizational culture levels for evidence-based practice before and after evidence application

The Evidence-Based Practice Organizational Culture Building Scale was used to interview nurses both before and after the implementation of the evidence. The findings demonstrated that there was a statistically significant difference between the baseline review and post-evidence application review scores of the Evidence-Based Practice Organizational Culture Questionnaire, which were (39.53 ± 7.62) and (82.63 ± 2.67), respectively (t = −23.864, *p* < 0.001).

## 4 Discussion

Clinical practice can be successfully standardized through evidence-based practice. Keep theory and practice in line, guide clinical practice with evidence (32), and consistently enhance outcome metrics (33). This study contained four guidelines, one national standard, one expert consensus, three evidence summaries, fourteen systematic assessments, and two RCTs. Following a thorough assessment of the literature and evidence, the scientific validity and efficacy of the evidence summaries were supported. As a result, clinical stakeholders participate in the decision-making process, integrating the evidence’s content with particular clinical settings to reach further conclusions regarding the necessity and viability of putting the evidence into practice (34). According to the baseline survey results, some evaluation indicators at the system level were zero, meaning that the medical staff in the anesthesia and perioperative medicine department had not given alarm fatigue management enough thought, that the department lacked pertinent procedures, systems, and standards, and that there was a low awareness rate of evidence-based knowledge. Consequently, the Evidence Application Team has created a checklist for managing and preventing alarm fatigue, an evaluation form for doing so, and an alarm assessment and handling procedure. Based on the review’s findings, the management system and hardware facilities were progressively enhanced, and interdepartmental collaboration and communication were reinforced in order to successfully lower alarm fatigue and raise the standard of nursing care. The implementation of the intervention, which included adjusting the alarm parameters, also led to an increase in workload. As a result, we also made adjustments to the distribution of manpower, hiring ten additional assistant nurses to boost staffing levels. To enhance operational consistency and advance evidence-based practice, alarm management-related procedures and systems were created and posted in terms of physical resources.

Among surgical patients, the frequency of alarm management-related adverse events decreased. The frequency of alarm-type adverse occurrences can be decreased by creating countermeasures for the four stages of the alarm lifecycle (35). The small sample size of the survey may have contributed to the lack of statistical significance in the comparison of the incidence of device operation and threshold on of the anesthesia machine and the incidence of device operation, lead wire connection, alarm on, and message display of the monitors before and after evidence application. There were statistically significant variations between the monitors’ threshold setting, technical operation, and alarm awareness prior to and following the application of evidence. Disparities in anesthesia machine technical functioning, message display, alert awareness, breathing line connection, and alarm on that were statistically significant, in agreement with the findings of Chen (36), who conducted a retrospective review of medical device adverse occurrences and determined that inadequate training was the primary cause, indicates the ongoing need for improved instruction in medical equipment management and alerting knowledge. Using a multimodal strategy that included organizational policy, alarm monitor training films, and nurse training sessions, Arkilic (37) discovered that alarm fatigue was significantly impacted by an increase in nursing staffing and the volume of data input from medical devices. In order to establish a departmental alarm assessment and handling procedure, we developed a checklist for alarm-related adverse occurrences, developed the countermeasures as a departmental system, and integrated them into the nursing and medical assessments. Evidence-based clinical practice effectively contributed to system standardization and work patterns, and improvement benefits were amplified at the system, healthcare, and patient levels.

Medical staff members’ knowledge and evidence-based practice skills in managing alarm fatigue were enhanced. The findings demonstrated a statistically significant difference between the anesthesiology health care providers’ scores on the Barriers to Evidence-Based Nursing Practice Scale and their scores on the current state of knowledge, beliefs, and behaviors regarding alert management before and after the implementation of the evidence, showing that nurses and anesthesiology practitioners have significantly improved their understanding of evidence-based practice and alert management. The department’s nurses had a weak evidence-based foundation, as seen by their low evidence-based practice skill score prior to the incorporation of evidence. This study established an online course to ensure the seamless implementation of evidence-based practice and to raise the level of knowledge regarding healthcare alert management and evidence-based practice ability. Personnel who have received systematic training and earned evidence-based related certificates, such as the Southern Hospital of Southern Medical University JBI Evidence-Based Nursing Training Certificate and the Australian JBI training certificate, were invited to give lectures.

## 5 Limitation

An important limitation of this study is that it is a study conducted in a single site, which is not well able to collect more data by crossing disciplines, and further multi-center studies with large samples are needed to confirm our findings. The non-synchronous before-after controlled trial design of this study necessitates the addition of patient-related outcome indicators, the use of more scientific research techniques for the subsequent quality improvement cycle, and the investigation of techniques and strategies to enhance quality and strengthen the improvement effect.

## 6 Implication for nursing management

The alarm fatigue and management of anesthesiologists and nurses were examined in this study. According to the study, the best evidence-based intervention for anesthesiologists’ and nurses’ alarm fatigue produced positive outcomes and enhanced their evidence-based skills. To optimize the alarm management capabilities of physicians and nurses in the PACU, it is recommended that anesthesiology managers focus on the management of alarm fatigue, provide frequent training, lower the number of false alarms, and support the use of intelligent technology.

## 7 Conclusion

To sum up, this study evaluated the clinical context, created strategies for improving implementation, analyzed obstacles to the application of the evidence before program implementation, and built review metrics based on an overview of the best evidence for managing and preventing alarm fatigue. The organizational culture has improved as a result of the evidence-based clinical application, which has raised the level of alarm knowledge, evidence-based practice, and healthcare implementation. It has also successfully decreased the incidence of unfavorable outcomes associated with anesthesia machine and patient monitor alarms. However, the clinical application of this best evidence has limitations. Because of the circumstances, some of the review indicators have not been clinically transformed. Future research will be grounded in the real-world clinical scenario to create appropriate and workable clinical strategies that will encourage the use of the best evidence in clinical practice and demonstrate its efficacy, as well as developing standardized guidelines.

## Data Availability

The raw data supporting the conclusions of this article will be made available by the authors, without undue reservation.
